# 

**Published:** 1894-01

**Authors:** 


### CONTENTS FOR JANUARY, 1894.

ORIGINAL COMMUNICATIONS. PAGE.

Empyema. By John Parmenter, M. D. .... 321
Some Interesting Cases from the Medical Service at the Cattaraugus County Almshouse. By Clarence King, M. D. .... 336

CLINICAL REPORT.

Clinical Memoranda from the Surgical Clinic at the Sisters' of Charity Hospital. By Herman Mynter, M. D. .... 341

PROGRESS IN MEDICAL SCIENCE.

Ophthalmology and Otology. By Alvin A. Hubbell, M. D. .... 345

SELECTIONS.

Observations on the Nature and Treatment of Angina Pectoris .... 351
The Treatment of Blepharitis Marginalis by Hydrogen Dioxid .... 354

EDITORIAL.

Football in Colleges .... 355
Topics of the Month .... 357

Personal .... 364
College Notes .... 365
Obituary .... 366
Society Meetings .... 366
Book Reviews .... 368
Miscellany .... 377
Literary Notes .... 380

